# Cognitive aging and dementia prevention: the time for psychology?

**DOI:** 10.18632/aging.204562

**Published:** 2023-02-27

**Authors:** David Bartrés-Faz, Cristina Solé-Padullés, Natalie L. Marchant

**Affiliations:** 1 Department of Medicine, Faculty of Medicine and Health Sciences and Institute of Neurosciences, University of Barcelona, Barcelona, Spain; 2 Institut d’Investigacions Biomèdiques August Pi i Sunyer (IDIBAPS), Barcelona, Spain; 3 Institut Guttmann, Institut Universitari de Neurorehabilitació adscrit a la Universitat Autònoma de Barcelona, Barcelona, Spain; 4 Division of Psychiatry, University College London, London, United Kingdom

**Keywords:** cognitive aging, psychological factors, dementia, prevention

Modifiable risk and protective factors (e.g. engaging in active lifestyles and avoiding alcohol or smoking amongst others) are seen as key agents for dementia prevention [1], and they also exert an important effect on cognitive trajectories of non-demented older adults [2]. Within this context, recent research has begun to identify psychological processes that confer relative risk and protection. For example, repetitive negative thinking (RNT), a cognitive process defined by self-relevant, persistent thoughts that elaborate on negative themes, has been associated with greater burden of typical Alzheimer’s disease (AD) pathological brain markers and accelerated cognitive decline over time [3]. In contrast, self-reflection [4], as well as purpose in life and other components of psychological well-being, may help to maintain cognition and boost cognitive resilience against neuropathological burden [5]. The possibility of incorporating psychological elements as key players in affecting one of the most important public health issues of the century opens a window of great therapeutic opportunity, particularly because fundamental psychological processes are at the core of cognitive-behavioural interventions that may help reduce dementia risk [6]. However, for this emergent area to develop and wield maximum benefit, major unanswered questions need to be addressed. Here, we highlight three main areas for future research.

**Figure 1:**
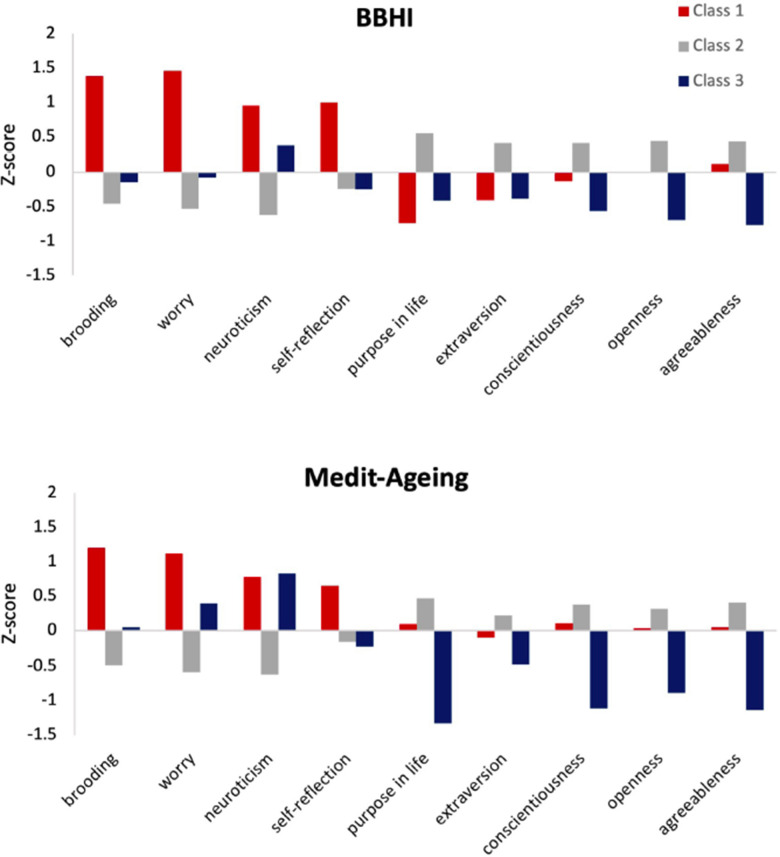
**Results from a latent profile analysis (LPA) in two independent European cohorts: the middle-aged *Barcelona Brain Health Initiative* (BBHI, N=741, mean age 53) and the older-adult *Medit-Ageing* (N=279, mean age 71) studies.** The figure shows strikingly similar latent profiles in both cohorts, each identifying a three-class solution: “high levels of psychological risk factors” (Class-1), “average levels of psychological risk and protective factors” (Class-2) and “low levels of psychological protective factors” (Class-3).

First, to date the effects of potential psychological risk and protective factors have been mainly reported in isolation. While helpful to identify key components, this approach cannot account for the complexity of psychological processes that may exist within a person, and how potentially competing processes may interact to impact on cognitive status, presence of biomarkers or brain integrity measures. To address this issue, we recently used a person-centered approach to define *psychological profiles* of participants. We found that individual psychological risk and protective factors aggregated into three identifiable psychological profiles which were consistent in two independent European cohorts (Figure 1). Future studies are needed to determine the nature of these different psychological profiles as being protective or risk factors. Such knowledge would contribute to more individualized psychological preventive therapeutic strategies.

Second, the current approach to consider risk and protective factors across multiple domains is reflected in major preventive clinical trials completed to date, where several domains (e.g. reducing vascular risk factors by improving nutrition or physical exercise) are targeted in parallel [7]. However, there is a lack of understanding about how psychological factors, or indeed profiles, integrate with other lifestyle factors across different groups of individuals. This is relevant, at least because: 1) engagement in behaviours that may confer risk (e.g. sedentary lifestyle, substance misuse) or protection (e.g. healthy diet, cognitively stimulating activities) may strongly modulate the impact of psychological factors on cognitive function, and 2) psychological factors may act as potent drivers of engagement in other healthy lifestyles. For example, related to the use of maladaptive stress coping strategies, individuals with high and persistent levels of rumination and worry might be more prone to social isolation and loneliness, which by themselves represent risk factors for dementia in advanced age. In this case, preventing social disconnection would be important, but additionally improving stress coping styles could be paramount. On the other hand, in the context of individuals with low engagement in protective lifestyles, the consideration of life purposes and meaningful values for the individual could lead to the identification and recommendation of new and alternative healthy behaviours, which the individual could potentially adhere to with high motivation. Developing further and systematic knowledge in this area, by using for instance health mobile technologies to monitor behaviour as a function of psychological profiles, could help in designing personalised, and ideally more effective, future interventions for habit change.

Finally, there is a need to explore brain mechanisms through which psychological processes may exert their protective or deleterious effects. While dysregulation of biological pathways associated with stress response (i.e. hypothalamic–pituitary–adrenal (HPA) axis) are frequently evoked in literature, our knowledge of systems-level brain network expression is only beginning to emerge. In this regard, and using resting-state functional magnetic resonance imaging (fMRI), a recent study from our group [8], investigated the associations between ratings of RNT and the functional connectivity of three main circuits; namely, the so-called triple network of attention. Overall, the default mode network from individuals with high RNT was less connected with the executive control network and more connected with the anterior salience network. Although speculative, these findings may indicate that top-down executive control systems are compromised amongst individuals with high RNT, perhaps underlying the previously reported cognitive decline associated with RNT [3], whereas on the other hand, connectivity between networks engaged in emotional processing (i.e. the anterior salience network) and introspective thought (i.e. default mode network) are accentuated. Another interesting aspect of this study is that the findings were observed amongst middle-aged healthy individuals, hence reflecting that associations between psychological processes and the status of brain networks can already be detected well before age-related accelerated cognitive changes or early manifestations of dementia may occur. However, many questions remain for future research. For example, is there a specificity of psychological risk and protective factors regarding their association with brain integrity metrics, compared to those related to clinical manifestations such as anxiety and depression symptoms? Recent literature suggests so, and for instance, the association between self-reflection and increased glucose metabolism in temporoparietal regions was observed even after adjusting for demographic variables, APOE status as well as depression [4]. Future research should also shed light in other key topics, such as whether psychological profiling may be useful to define populations most likely to benefit from preventive interventions, or if complementing the standard assessment of psychological processes (e.g. questionnaires) using a more fine-grained approach, for example with real-time evaluations during experimental studies, would improve our sensitivity to detect associations with cognition and dementia biomarkers.

In summary, we propose that with momentum gathering, now is the time for psychology to make important contributions to cognitive ageing and dementia prevention research. More refined characterization of psychological processes that may confer relative risk or protection, improved understanding of their interplay with other modifiable factors and of their biological substrates, and their consideration and incorporation into future clinical trials are all critical avenues to pursue. Progress in this area should lead to the creation and implementation of a psychological framework, with operational definitions, that would allow researchers and clinicians to work together to promote healthy cognitive ageing and dementia prevention.
